# Supplementary material for: Novel p-Hydroxybenzoic Acid Derivative Isolated from Bacopa procumbens and Its Antibacterial Activity
Source: Antibiotics (Basel). 2025 Jun 7;14(6):591. doi: 10.3390/antibiotics14060591 (PMC12190013; doi:10.3390/antibiotics14060591)
Supplement: Supplementary file 1 [file antibiotics-14-00591-s001.zip › antibiotics-3645279-supplementary.pdf]

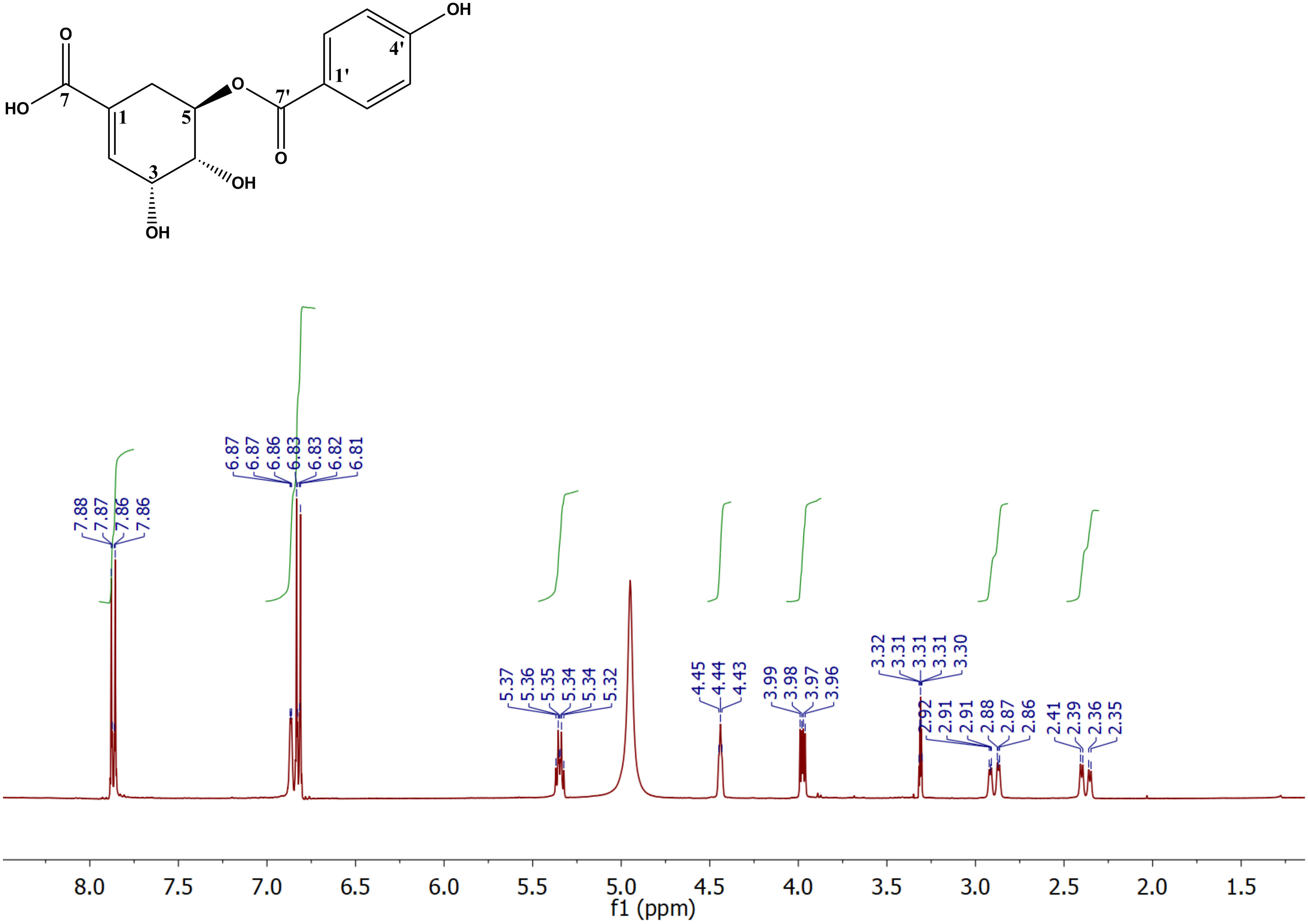

Fig. S1. 1H-NMR (CD3OD, 400 MHZ) compound 5-(p-hydroxybenzoyl) shikimic acid (5pHSA)

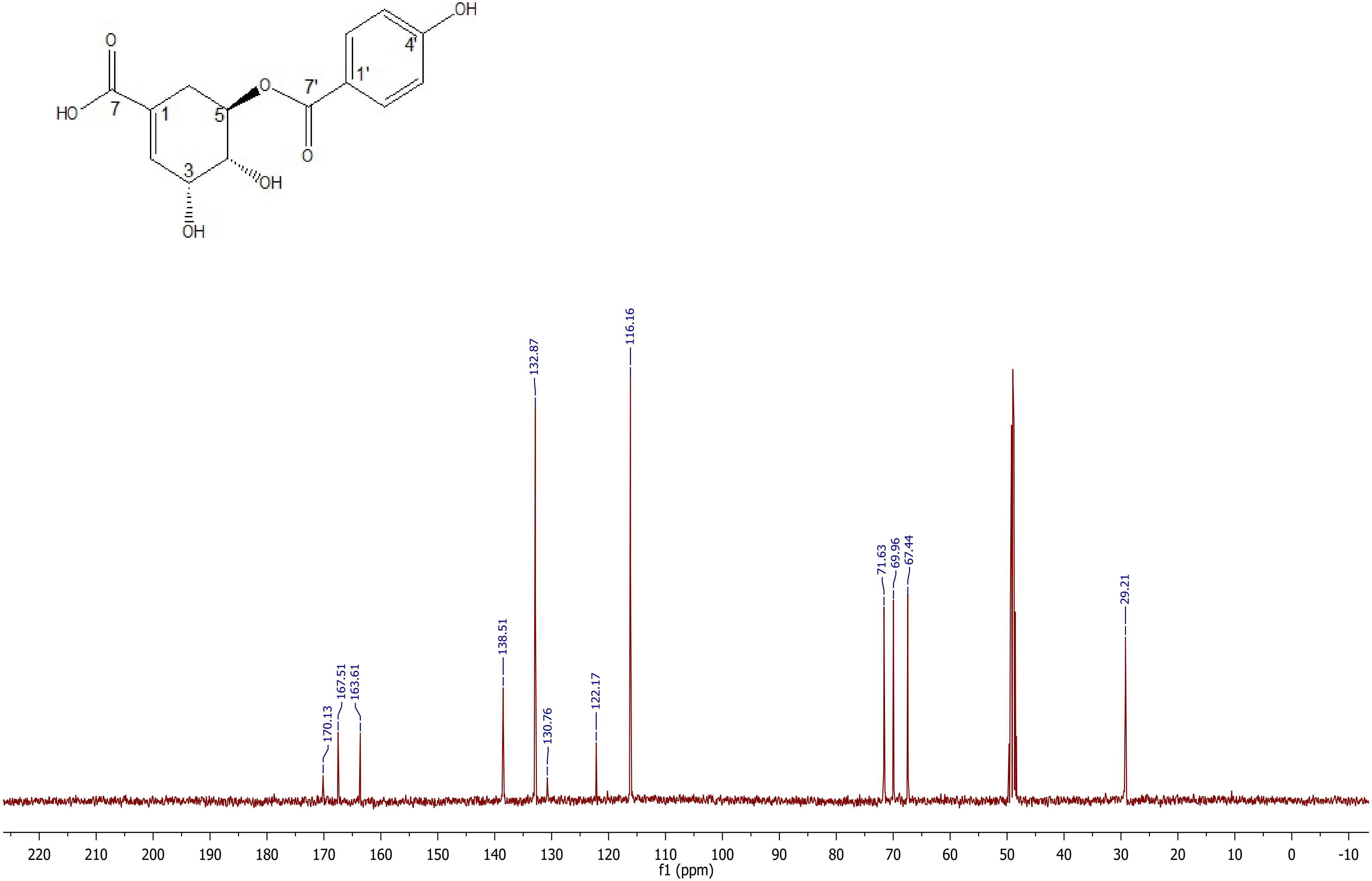

Fig. S2 . ^{13}C-NMR (CD_3OD, 100 MHZ) compound 5-(p-hydroxybenzoyl) shikimic acid (5pHSA)

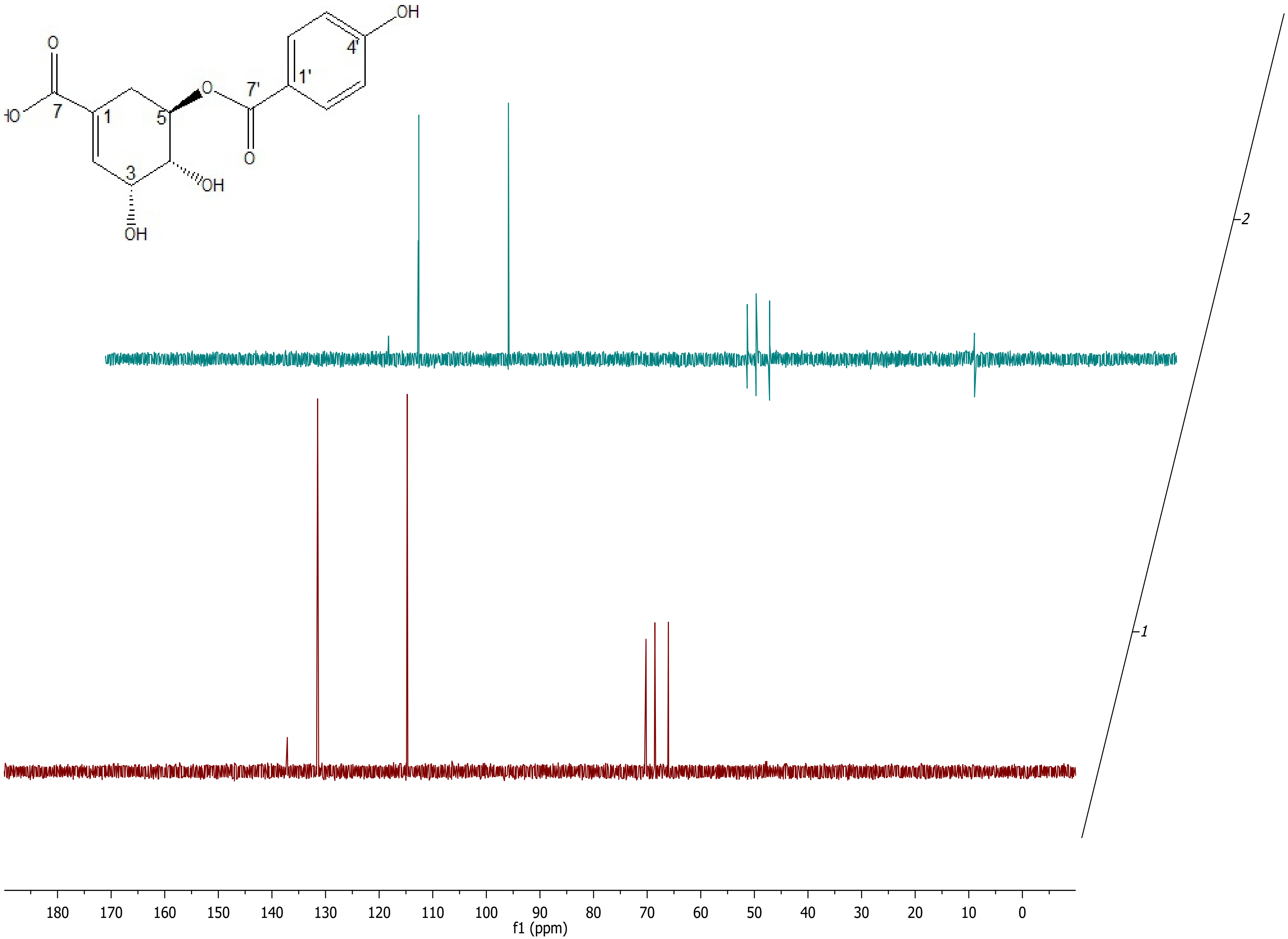

Fig. S3 . ^{13}C(DEPT)-NMR (CD_3OD, 100 MHZ) compound 5-(p-hydroxybenzoyl) shikimic acid (5pHSA)

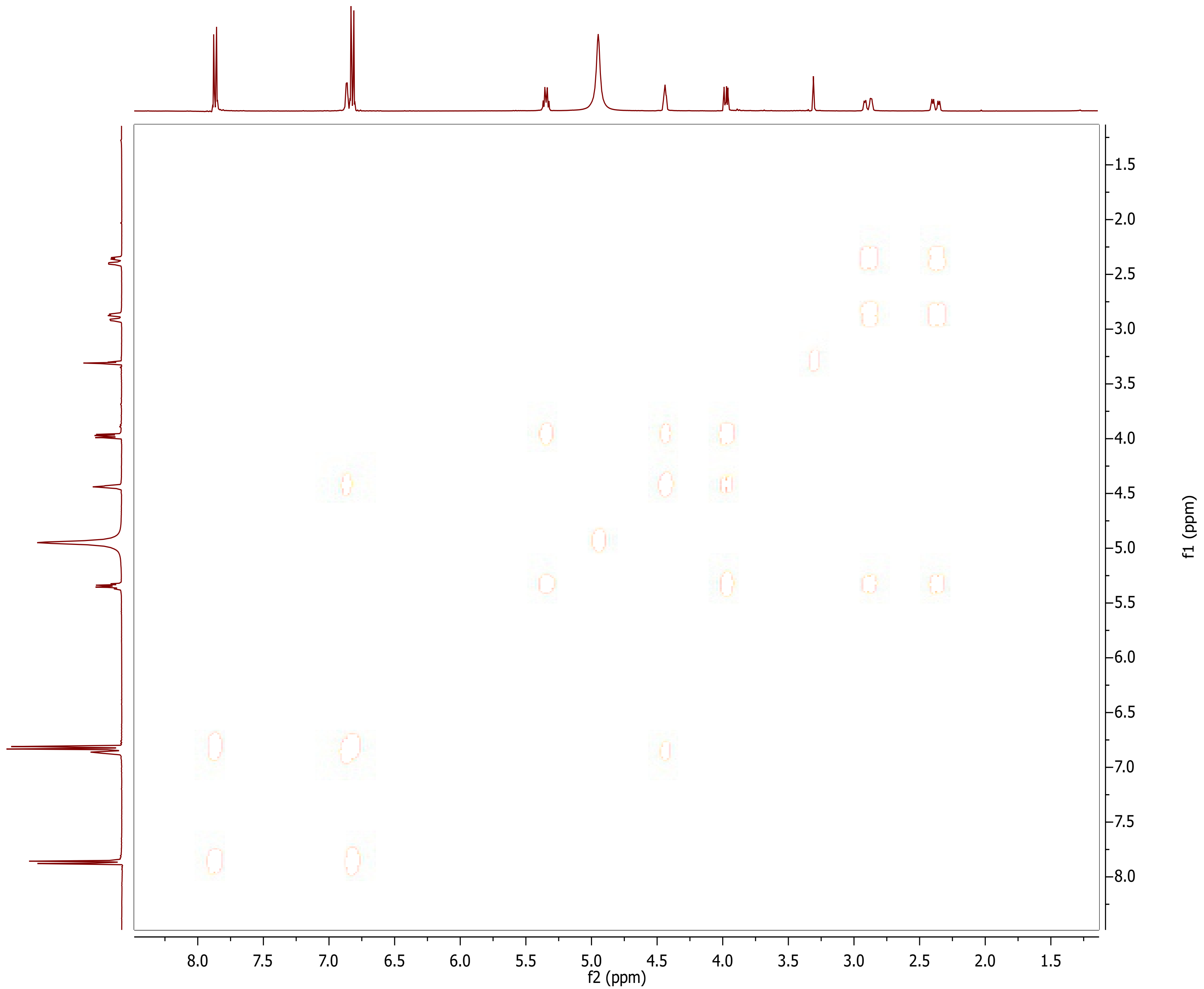

Fig. S4 . ^1H-^1H(COSY)-NMR (CD_3OD, 600 MHZ) compound 5-(p-hydroxybenzoyl) shikimic acid (5pHSA)

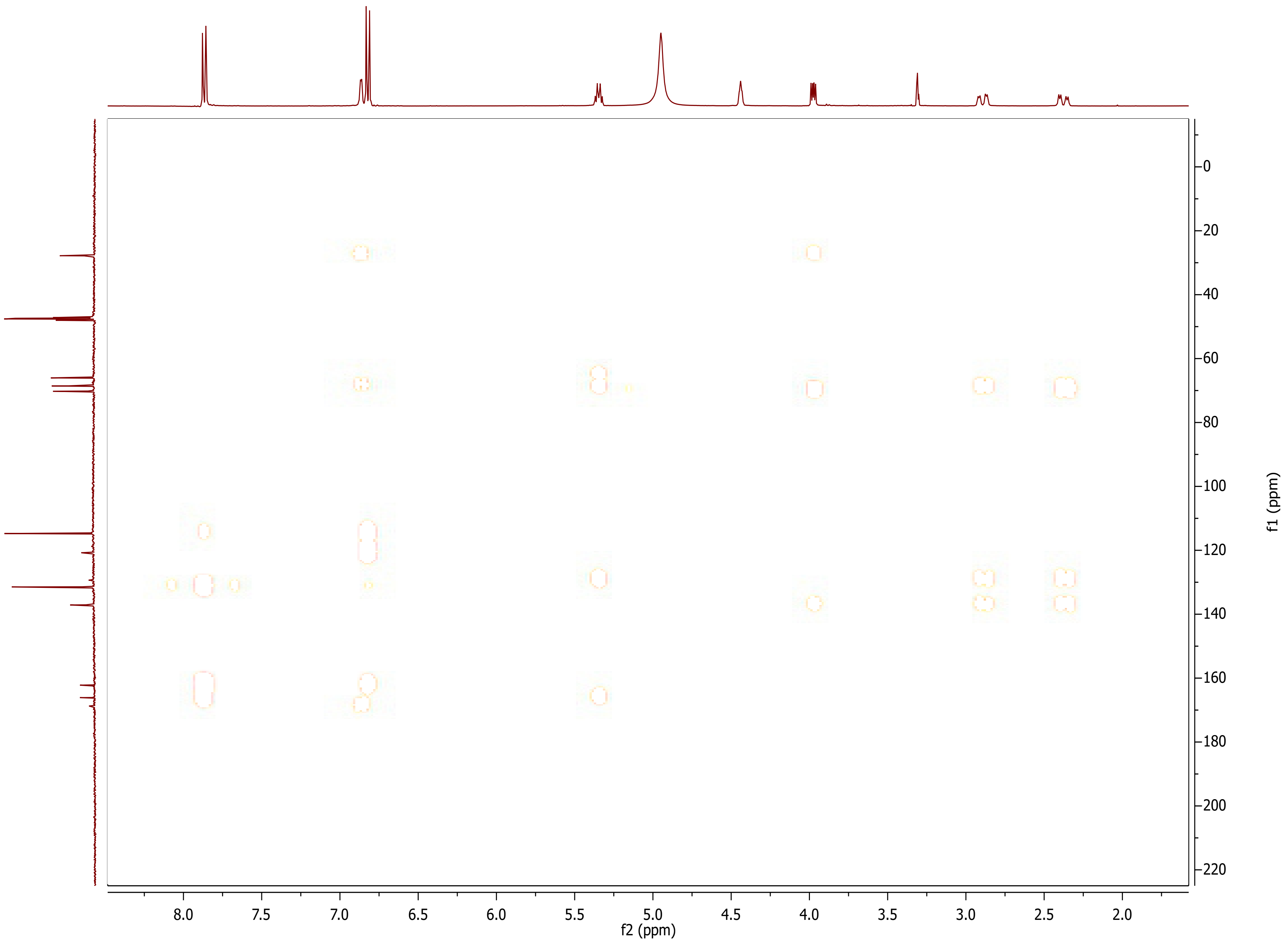

Fig. S5 . ^{1}H-^{13}C(HSQC)-NMR (CD_3OD, 600 Mhz) compound 5-(p-hydroxybenzoyl) shikimic acid (5pHSA)

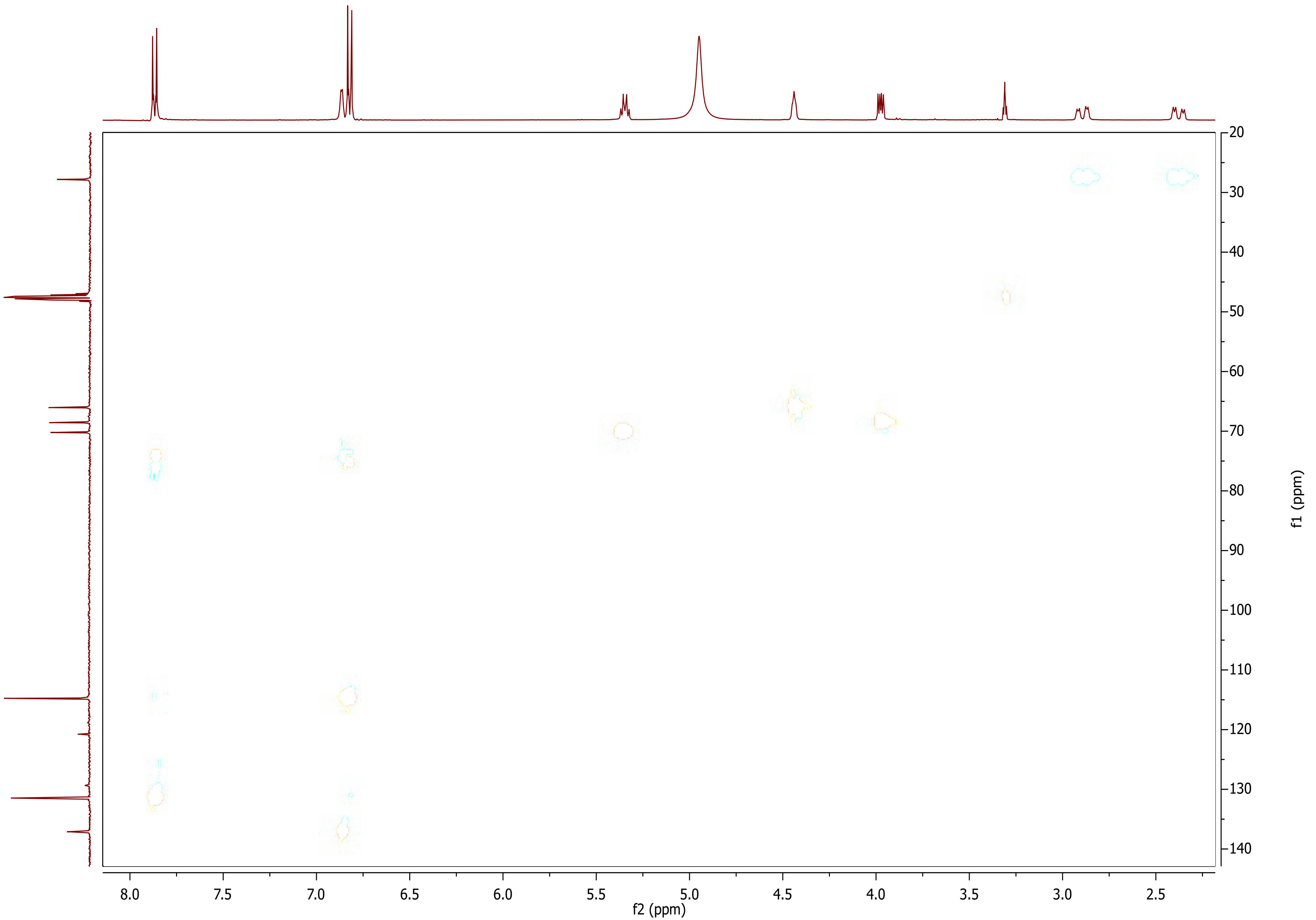

Fig. S6 . ^{1}H-^{13}C(HSQC)-NMR (CD_3OD, 600 MHz) compound 5-(p-hydroxybenzoyl) shikimic acid (5pHSA)

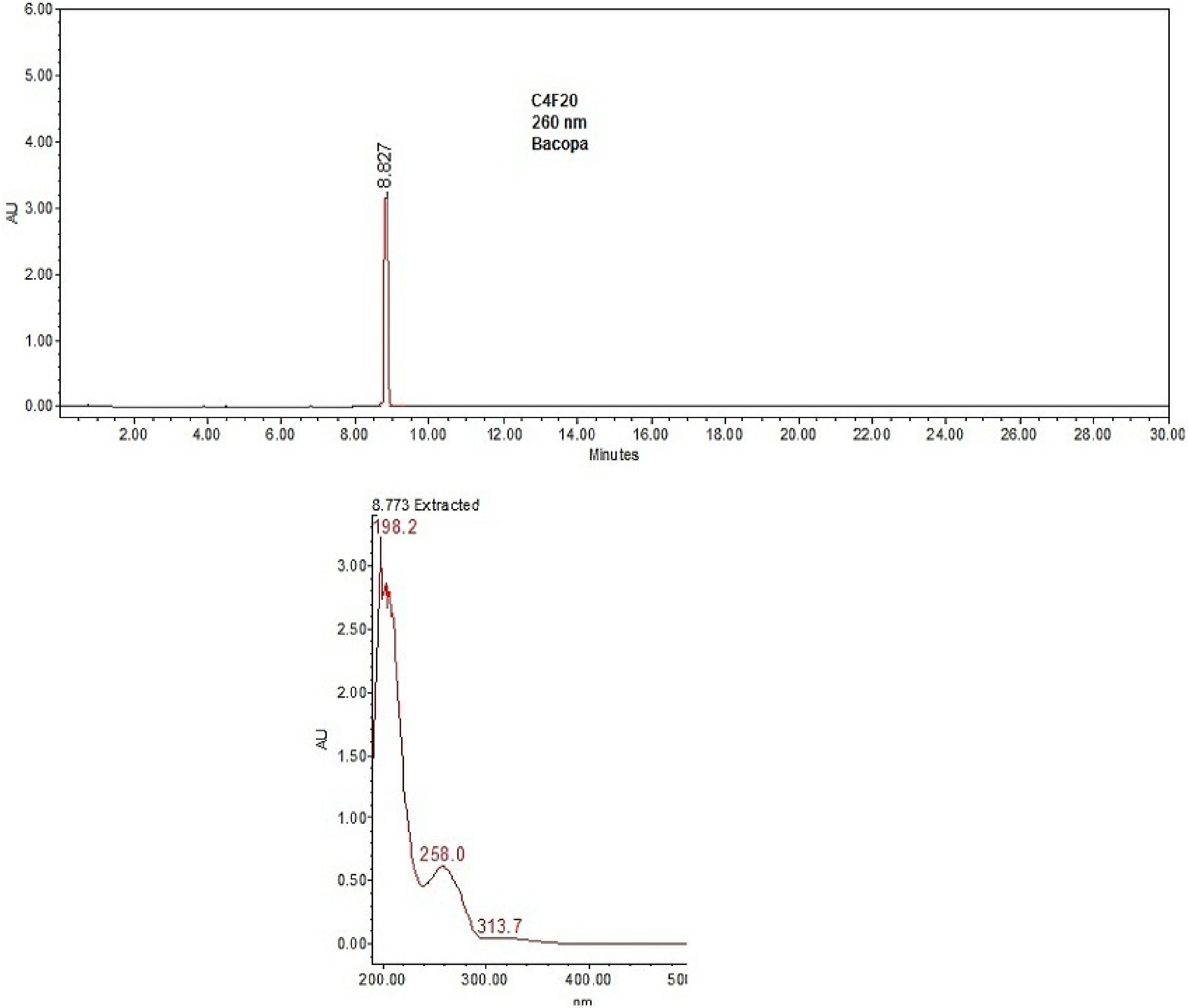

Fig. S7． HPLC chromatogram and UV spectrum of compound 5-(p-hydroxybenzoyl) shikimic acid (5pHSA)

a)

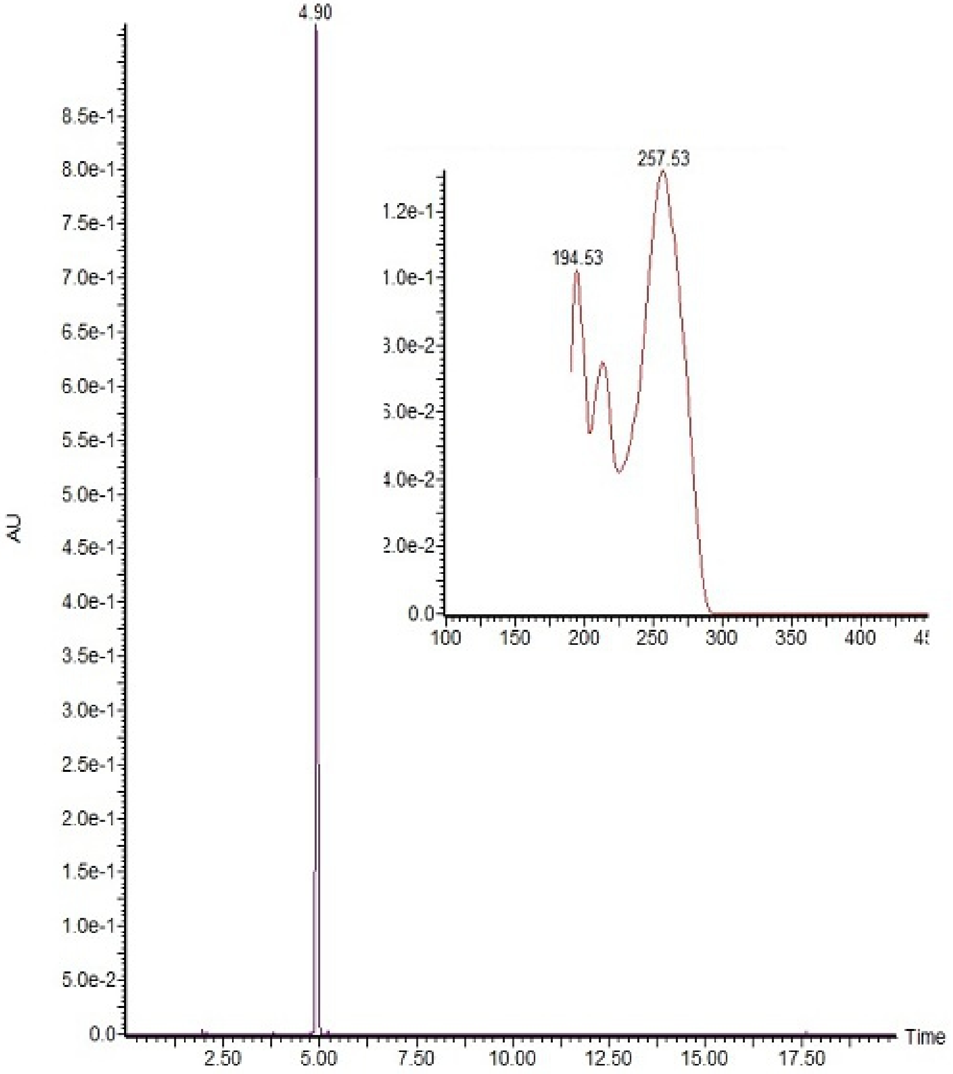

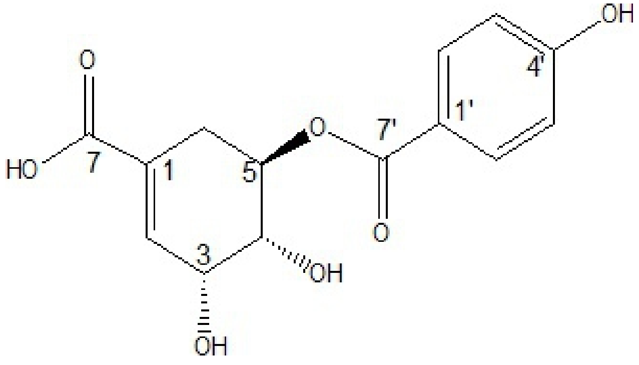

m/z 294.07 $[M-H]^-$

b)

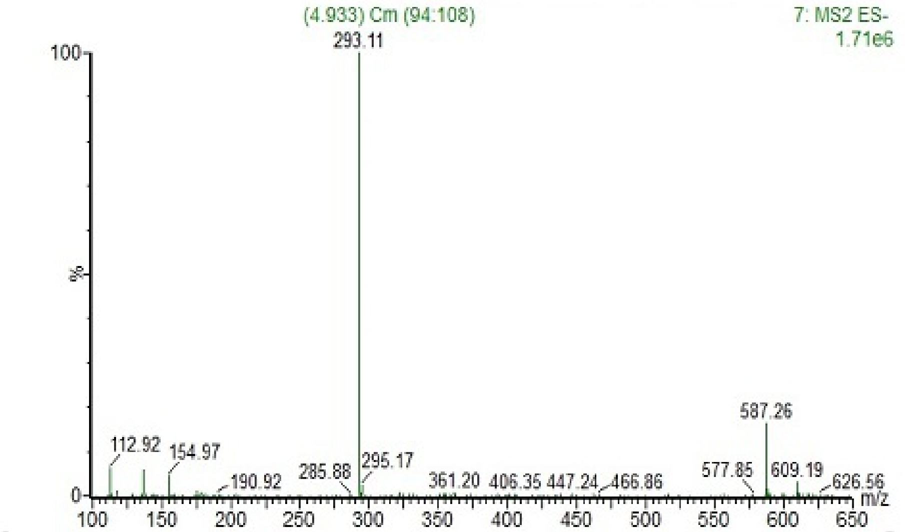

Figure S8. a) UPLC-mass spectrometry chromatogram and UV spectrum and b) mass EI spectrum of 5-(p-hydroxybenzoyl) shikimic acid (5pHSA).

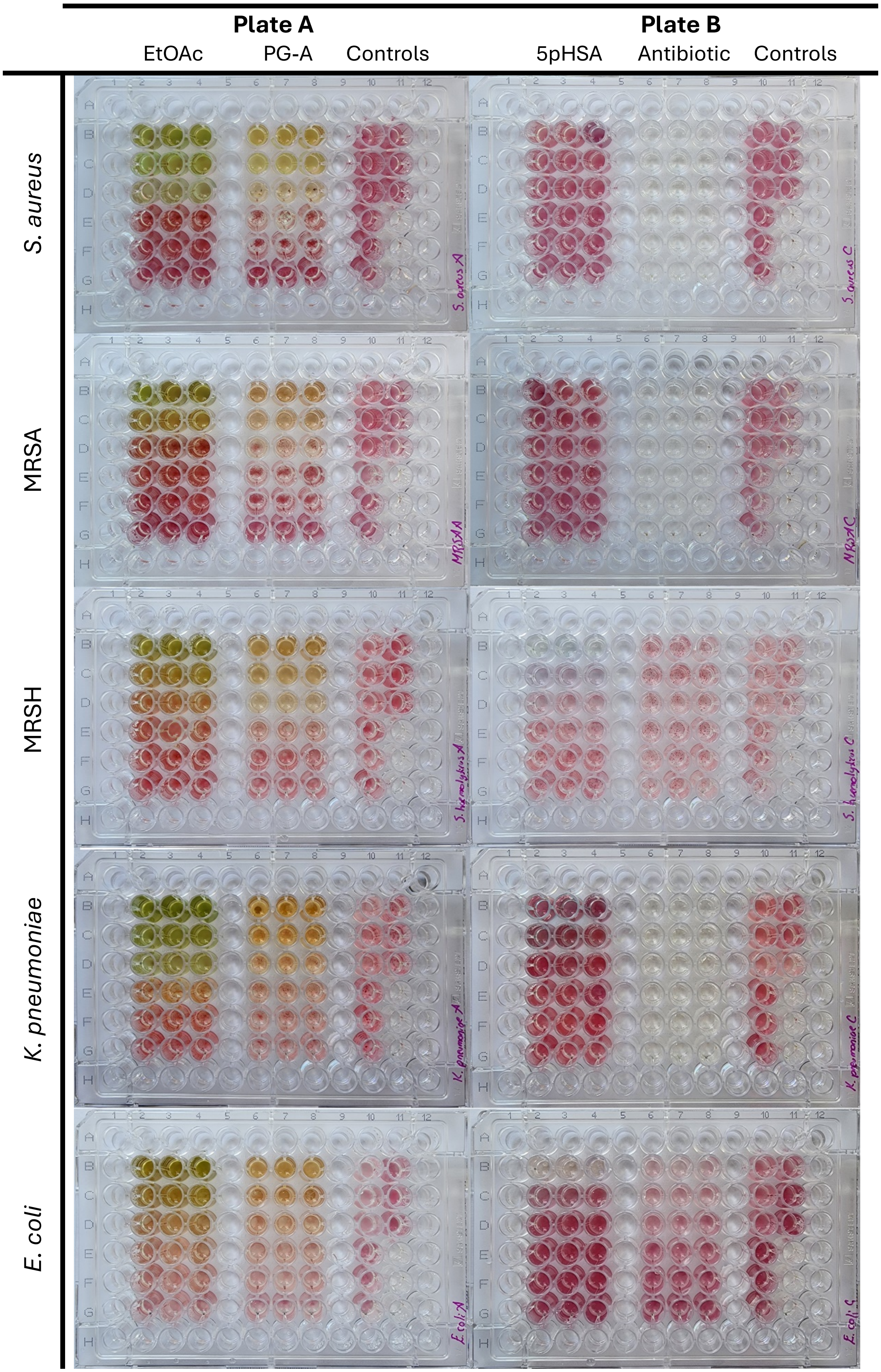

Fig. S9. Antimicrobial test microplates: EtOAc - Ethyl acetate fraction, PG-A – ProcumGastrodin A, 5pHSA - 5-(p-hydroxybenzoyl) shikimic acid, Antibiotic – Clarithromycin, Controls: sterilized water, 20% DMSO solution, bacteria inoculum as positive control and Mueller-Hinton broth as blank.
